# The direction of word stress processing in German: evidence from a working memory paradigm

**DOI:** 10.3389/fpsyg.2014.00574

**Published:** 2014-06-11

**Authors:** Frank Domahs, Marion Grande, Walter Huber, Ulrike Domahs

**Affiliations:** ^1^Klinische Linguistik, Institut für Germanistische Sprachwissenschaft, Philipps-Universität MarburgMarburg, Germany; ^2^RWTH Aachen University HospitalAachen, Germany; ^3^University of CologneCologne, Germany

**Keywords:** lexial stress, directionality, reading, non-words, pseudowords, correlation

## Abstract

There are contradicting assumptions and findings on the direction of word stress processing in German. To resolve this question, we asked participants to read tri-syllabic non-words and stress ambiguous words aloud. Additionally, they also performed a working memory (WM) task (2-back task). In non-word reading, participants’ individual WM capacity was positively correlated with assignment of main stress to the antepenultimate syllable, which is most distant to the word’s right edge, while a (complementary) negative correlation was observed with assignment of stress to the ultimate syllable. There was no significant correlation between WM capacity and stress assignment to the penultimate syllable, which has been claimed to be the default stress pattern in German. In reading stress ambiguous words, a similar but non-significant pattern was observed as in non-word reading. In sum, our results provide first psycholinguistic evidence supporting leftward stress processing in German. Our results do not lend support to the assumption of penultimate default stress in German. A specification of the lemma model is proposed which seems able to reconcile our findings and apparently contradicting assumptions and evidence.

## INTRODUCTION

How do we know which syllable of a polysyllabic word should receive main stress? In figuring out – should we start from the beginning (i.e., left edge) or from the end (i.e., right edge) of the word? The answer seems easy in languages with fixed stress position: We should start from the left edge in languages with fixed stress on the first (e.g., Cahuilla, Hungarian, and Icelandic), second (e.g., Dakota, Mapudungun, and Tolai), or third (e.g., Winnebago) syllable, while we should start from the right edge in languages with fixed stress on the ultimate (U, e.g., Balinese, Persian, and Weri), penultimate (PU, e.g., Djingili, Polish, and Quechua), or antepenultimate (APU, e.g., Greek, Macedonian, and Paumari) syllable (for an overview see Goedemans and van der Hulst, 2014). The matter is less obvious in languages with variable stress (e.g., English, German, and Russian). As in those languages the position of main stress is largely unpredictable, it has been suggested that this information has to be stored in the mental lexicon for all words. However, it is not clear, whether, for instance, the lexical entry of the German word *Veránda* codes main stress position as second or prefinal – in other words, whether retrieval of stress position proceeds in a rightward or leftward manner (or with no specific directionality at all).

Based on regularities or analogies generated from their lexical knowledge, even speakers of languages with unpredictable stress are able to assign stress to non-words (Janssen, 2003b; Tappeiner et al., 2007; Röttger et al., 2012; Domahs et al., 2014). Typically, the assignment of stress to non-words is characterized by large interindividual variance. Moreover, the assignment of stress to both existing words and non-words may leave behavioral traces of processing demands. The present study aims to explore the interaction of interindividual variance in stress assignment and specific computational demands for different stress positions to investigate the direction of stress processing in German. In the remainder of this section, we will first summarize arguments on the direction of stress computation in German and then outline the rationale of the study.

### THE DIRECTION OF STRESS COMPUTATION GERMAN

The computation of main stress position may, in principle, start from the beginning or from the end of the word (i.e., rightward or leftward assignment, respectively). There are arguments for both options in German, which will be reviewed in the following.

Most current accounts on German stress assignment – explicitly or implicitly – proceed from the assumption that the syllable to be assigned main stress is defined in a leftward fashion, starting from the right edge of a word. This holds true irrespective of whether these accounts opt for quantity-sensitive or for quantity-insensitive stress assignment. Quantity-sensitive accounts state that the structure or weight of the final and/or prefinal syllable is a particularly important predictor of the position of main stress in German (Vennemann, 1991; Féry, 1998; Domahs et al., 2008). Quantity-insensitive accounts typically assume that the PU is the default stress position in German, all other stress patterns being exceptions which require lexicalization (Eisenberg, 1991; Wiese, 1996). Leftward stress computation in German is supported by the fact that only one of the last three syllables (APU, PU, or U) can bear main stress (*“three-syllable window,”* Giegerich, 1985; Vennemann, 1991; Zonneveld et al., 1999). Psychologically, the three-syllable window seems to be very robust. It was, for example, obeyed in a patient with acquired language impairment, who otherwise showed severe phonological and prosodic deficits (Janßen and Domahs, 2008).

One major argument for rightward stress computation comes from the psycholinguistic “lemma” model of speech production developed by Levelt et al. (1999). In this model, a metrical frame is retrieved (independently from the sequence of phonemes) which determines the number of syllables and the position of main stress in case of non-default stress assignment. In a further processing step, which is called prosodification, the metrical frame is filled with segments and – in the case of default assignment – the stress position is assigned. Crucially, first syllable stress is assumed to be the default in German (as in Dutch and English). In fact, evidence reported by Schiller et al. (2006) seems to lend support to such a rightward processing of metrical stress in Dutch: In a monitoring task, subjects were faster to detect stressed syllables at the beginning compared to stressed syllables at the end of words which they had to name implicitly from pictures. Yet, these authors themselves note that their observation may also be caused by the incremental (i.e., rightward) functioning of the monitoring system rather than by the incremental functioning of stress processing itself. Note that the assumption of first syllable stress as default in German as implemented in the lemma model (Levelt et al., 1999), although conceptually in clear contrast to the assumption of a PU default in German (Eisenberg, 1991; Wiese, 1996), makes identical predictions for the huge bulk of existing words, given that most monomorphemic word types in the German corpus consist of one or two syllables. In trisyllabic words, however, the predictions based on leftward computation differ from the predictions of the lemma model.

There is a third set of accounts, which assume that there are two co-phonologies of German with different implications for the direction of stress computation. According to those accounts, the default position of main stress in native German words is the first syllable, whereas stress in non-native words would be computed in a leftward manner starting from the right edge of the word (Wurzel, 1970, 1980; Benware, 1980; Féry, 1986). However, a number of authors disagree with the need to distinguish between native and non-native German phonology (Giegerich, 1985; Hall, 1992; Wiese, 1996).

### THE PRESENT STUDY

In sum, the question in which direction metrical stress is computed in German is still open. In our experiment, we explored the possibility that the processing of word stress in German occurs in a leftward instead of a rightward fashion, as predicted by a number of different phonological theories (Eisenberg, 1991; Vennemann, 1991; Wiese, 1996; Féry, 1998; Domahs et al., 2008). Note that we are taking a cognitive perspective here, rather than a purely descriptive linguistic approach. In this cognitive perspective, different stress positions may be associated with different computational costs. Specifically, processing costs, operationalized as working memory (WM) load, should increase with increasing distance of stress position from the starting point of computation (left or right edge of the word). If stress computation works from right to left, then computational WM load should increase in the following direction: U < PU < APU stress position. The opposite hierarchy is expected in the case of rightward stress computation. For instance, the assignment of stress to the first and the final syllable in non-words with a VC.V.VCC^1^ structure (e.g., *Rulkomenk*) is approximately balanced across participants in group analyses (43 and 47%, respectively), while the second syllable is only rarely stressed (Janssen, 2003b). However, if the computation of stress operates, indeed, in a leftward fashion, then it requires additional processing steps to identify and stress the APU position compared to placing stress on the U position, i.e., APU stress assignment is computationally more demanding than U stress assignment. Consistent with the right-to-left hypothesis, two patients with reduced WM span (Janssen, 2003a; Janßen and Domahs, 2008) produced virtually no APU stress on pseudowords, while a group of healthy subjects produced up to 50% of this stress pattern with the same material. More generally, the existence of the three-syllable window in German may be interpreted as consequence of leftward stress assignment subject to processing limitations.

To pursue our hypothesis, we examined non-word reading of native speakers of German whose WM capacity was quantified using a 2-back task (Zimmermann and Fimm, 1993). The use of non-words not only avoids the influence of lexical variables (e.g., word frequency) as far as possible but also ensures that the stress position has to be computed instead of retrieved from long-term memory (i.e., the mental lexicon).

We wanted to use the fact that there is a large degree of interindividual heterogeneity in word stress assignment, at least partly related to WM (Heisterueber et al., 2014). Specifically, it was predicted that the proportion of computationally complex APU stress assignment across stimuli should be positively correlated with the individual WM capacity. In other words: the more limited the WM capacity the fewer computationally complex stress assignments should be observed.

Participants were also asked to read a short story containing words, which can be stressed on different syllables (i.e., stress ambiguous words). Given that German is a language with largely unpredictable stress, the position of main stress should be lexicalized for these words. However, it may still be that a participant’s WM capacity influences his/her preferred stress position for such words. This influence may be less strong than the one expected for non-words, as the computational impact of stress position may be less pronounced in lexical retrieval than in actual computation of a stress pattern.

Some accounts of stress assignment in German assume that tri-syllabic words with a closed final syllable are parsed into two metrical feet (a final non-branching foot and a preceding binary one ([σσ)_F_(σ)_F_]_ω_), while words with an open final syllable are only parsed into one foot ([σ(σσ)_F_]_ω_), leaving an unparsed initial syllable, where stress assignment is disfavored (Alber, 1997; Domahs et al., 2008; Knaus and Domahs, 2009). Based on this analysis, words with a closed final syllable have more potential landing sites for main stress than words with open final syllable.

Note that the potential existence of a default stress pattern may overwrite the effect of computational direction. In this case, it may be that the default stress assignment is computationally easier than stress assignment to other positions. Potential default stress positions in German are the first syllable (Levelt et al., 1999) or the PU (Eisenberg, 1991; Wiese, 1996).

In sum, we want to make use of interindividual variance in cognitive processing capacity to distinguish between easier and more difficult stress positions indicative of the direction of stress computation in German.

## MATERIALS AND METHODS 

### PARTICIPANTS

Participants were recruited from retirement homes in the city of Aachen (Germany) and the orthopedic ward of the RWTH Aachen University Hospital. Thirty-eight participants performed a reading task with a list of 60 existing German words (20 words with APU, PU, and U stress pattern, respectively, in randomized order, see Tappeiner et al., 2007). Two participants read less than 80% correct and were excluded from further analyses. In the remaining sample, there were 20 women and 16 men. All participants were native speakers of German, coming from a heterogeneous educational background (6 had obtained German *Abitur*, 19 had finished *Realschule*, and the remaining 11 had finished *Hauptschule*). All but two participants were right handed according to their own disclosure.

Participants were aged between 52 and 94 years (mean = 72.1). This age range was chosen to increase the interindividual variance in WM capacity (Dobbs and Rule, 1989; Brockmole and Logie, 2013; Murre et al., 2013). No participants with diagnosed dementia or neurological illness were included. It was made sure that all participants used their glasses and/or hearing aid if necessary. All participants gave their informed consent and received a compensation of 5 Euros. The study was approved by the Institutional Review Board of the Medical Faculty at RWTH Aachen University (EK 182/06).

### TASKS

Participants performed three tasks: a non-word reading task, a reading task with stress-ambiguous existing words, and a 2-back task.

We used the non-word reading task designed by Janssen (2003b; see also Domahs et al., 2014). In this task, non-words have to be produced within a carrier sentence, to prevent from artifacts due to reading isolated non-words in a list. The carrier sentence was always the same throughout the task (*Ich habe gehört, dass Peter … gesagt hat*. [I have heard that Peter said …]) to control for interference from sentence prosody. Participants were instructed to first read the non-word silently and only if they felt ready to produce it fluently to utter the carrier sentence containing the target-non-word.

In the second task, participants were asked to read a small purpose-made story (33 lines) containing 8 existing words which are stress ambiguous in German (see Stimuli). Target words were not highlighted in the text and participants were unaware of the specific purpose of this task (i.e., they were globally instructed to read the story aloud).

The 2-back task is a supplement to a larger battery testing attentional functions (TAP, Zimmermann and Fimm, 1993). Participants see a sequence of isolated letters on a screen, in a self-paced speed of presentation. They are asked to indicate by a button press, whether any given letter in this sequence is identical to its pre-predecessor. (e.g., A–E–C–E–K–L–K, required yes-answers highlighted). Thus, this demanding task requires a variety of executive or WM functions including storing and updating of relevant information and inhibition of irrelevant information. As a kind of shorthand term, we will refer to the underlying construct tested with the 2-back task as WM capacity. Note that in this task the position of elements within a sequence is crucially important.

### STIMULI

In the non-word reading task, we used the set of stimuli described by Janssen (2003b, see also Domahs et al., 2014). These are phonotactically legal three-syllabic non-words in eight syllable structure conditions (rhyme structures: VC.V.VCC, V.VC.VCC, VC.V.VC, V.V.VC, V.VC.VC, V.V.V, V.VC.V, and VC.VC.V). These eight conditions were designed to examine the role of syllable structure on stress assignment, particularly focusing on the weight of the final syllable, as this seems to be most influential (Janssen, 2003b; Röttger et al., 2012; Domahs et al., 2014). However, the stimulus set did not include all logically possible combinations of syllable structures. Conditions with three heavy syllables (VC.VC.VC and VC.VC.VCC) were excluded, because such words are not attested in German. Furthermore, words with super-heavy syllables and light penult and antepenultimate (V.V.VCC) as well as with light final and penult and heavy antepenult (VC.V.V) were not tested, because such conditions would probably not add further insights into the role of quantity on stress assignment. In the item construction, resyllabifications of coda consonants as onset consonants of the following syllable were avoided by filling each onset position. In addition, in syllable contacts the sonority of consonants was chosen such that the parsing of consonants into complex onsets was made unlikely (e.g., a non-word like bat.ram could be syllabified as ba.tram, while las.fon.ta cannot be syllabified as *la.sfon.ta). Potential similarities to existing words were avoided as far as possible by including only items whose final two syllables did not rhyme with existing words. For further details on stimulus selection, see Domahs et al. (2014).

There were 10 items per condition, 80 items overall, which were presented in pseudorandomized order, interspersed with 40 one- and two-syllable filler non-words as well as 13 four-syllable non-words to prevent participants from using an individual “default” stress pattern consistently across the whole list of items. Although in general the target non-words lead to different specific stress assignment preferences depending on their syllable structure (e.g., words with V.VC.V structure are preferably stressed on PU syllable), there is always a large degree of interindividual variance – which so far is left unexplained – such that in no condition non-words are exclusively stressed on one syllable (Janssen, 2003b; Tappeiner et al., 2007; Röttger et al., 2012).

In the word reading task, eight target words embedded in a short story were stress ambiguous, i.e., they can receive either APU or U stress in German (*Kabarett, Telefon, Mikrofon, Dromedar, Marzipan, Alkohol, Megafon, Horizont*). Stress ambiguity was confirmed by the Duden^®^ online dictionary (http://www.duden.de). The fact that most stress ambiguities in German involve APU vs. U main stress position can be accounted for by the similarity of their underlying foot structure and the dissimilarity of the underlying PU foot structure and by the related fact that only words with APU and U stress consist of two metrical feet and therefore allow for stress variance (Domahs et al., 2008, 2013; Janßen and Domahs, 2008; Röttger et al., 2012). Note that preference for a specific variant of stress ambiguous words largely depends on the speaker’s regional variant of German. Other possible sources of interindividual variance, in particular WM capacity, have not been reported so far.

### ANALYSES

Participants’ oral responses were recorded and transcribed later by a trained speech-language therapist who was blind to the hypotheses of the experiment. Main stress was determined based on perceptual judgment^2^. In cases of any uncertainty, transcription was discussed with an experimenter. If no consensus could be obtained for a specific item, this item was excluded from analyses.

In non-word reading, only responses without segmental errors for which main stress position could be identified unambiguously were included in the analyses. These criteria were fulfilled by 90.8% of the given responses. Dependent variables were the proportions of APU, PU, and U stress assignment in the target non-words. Note that these proportions are interdependent.

In word reading, the dependent variable was the proportion of APU stress assignment in the target words. Note that the proportion of U stress assignment is complementary with the proportion of APU stress assignment. There were 94.1% analyzable word items.

In the 2-back task, the dependent variable (*“WM capacity”*) was the number of correct yes-responses (max = 14).

Given the non-parametric nature of the data, we explored the relationship between individual WM capacity on the one hand and the proportion of stress patterns in non-word and word reading on the other using Spearman’s rank correlation coefficient.

## RESULTS

### NON-WORDS

**FIGURE 1:**
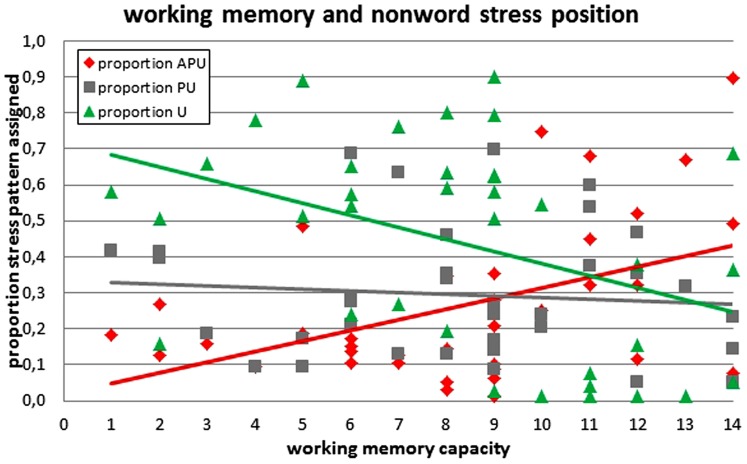
**Assignment of main stress in non-word reading as a function of individual working memory capacity.** (Note that we plotted linear functions in this graph, while we used a non-parametric procedure in actual analyses, which does not assume a linear function.)

We found a significant positive correlation between individual WM capacity and the proportion of APU stress assigned (*r*_S_ = 0.344, *p* = 0.040) and a (complementary) negative correlation between WM capacity and the proportion of final stress assigned (*r*_S_ = –0.427, *p* = 0.009; see **Table 1**, **Figure 1**). There was no significant correlation between WM capacity and the proportion of PU stress assigned (*r*_S_ = –0.081, *p* = 0.637).

**Table 1:** Results as a function of structural conditions.

		Correlations			Proportion (%)			
	Condition	APU	PU	U	APU	PU	U	Not analyzable
**1**	VC.V.VCC	0.393*	–0.133	–0.316	30.8	14.2	42.8	12.2
**2**	V.VC.VCC	0.535***	0.084	–0.404*	21.2	24.4	42.8	11.7
**3**	VC.V.VC	0.357*	0.078	–0 369*	33.9	13.3	43.9	8.9
**4**	V.V.VC	0.385*	–0.176	–0.353*	36.1	13.9	42.8	7.2
**5**	V.VC.VC	0.282	–0.082	–0.243	20.3	24.4	44.2	11.1
**6**	V.V.V	0.319	–0.125	–0.190	17.5	43.1	33.6	5.8
**7**	V.VC.V	0.046	0.184	–0.304	15.0	42.8	32.5	9.7
**8**	VC.VC.V	0.009	0.104	–0.361*	17.5	43.9	31.4	7.2
	Total	0.344*	–0.081	–0.427**	24.0	27.5	39.2	9.2

Correlations are indicated as Spearman‘s rank correlation coefficients between WM capacity and proportion of stress position assigned. Significant correlations are marked with *p ≤ 0.05, **p ≤ 0.01, or ***p ≤ 0.001, respectively.

The pattern of correlations was consistent across conditions (i.e., positive for APU, negative for U, and non-significant for PU). However, looking at the influence of syllable structure (possibly indicative of foot structure), significant correlations were almost exclusively found for non-words with closed final syllable, which – due to their foot structure – offer more potential landing sites for main stress than non-words with open final syllable (see **Table 1**, conditions 1–5).

Moreover, there was increasing interindividual variance of APU stress assignment with increasing WM capacity, i.e., there were increasing absolute residuals from a linear function (*r*_S_ = 0.352, *p* = 0.035). There were no such significant relationships between WM capacity and the variance of either PU (*r*_S_ = 0.045, *p* = 0.796) or U (*r*_S_ = 0.260, *p* = 0.126) stress assignment.

### STRESS AMBIGUOUS WORDS

**FIGURE 2:**
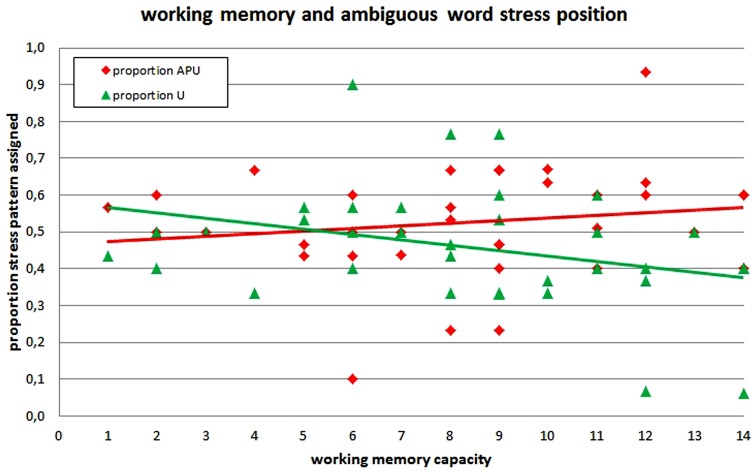
**Assignment of main stress in stress ambiguous words as a function of individual WM capacity.** (Note that we plotted linear functions in this graph, while we used a non-parametric procedure in actual analyses, which does not assume a linear function.)

There was a near-significant negative correlation between WM capacity and the proportion of final stress assigned (*r*_S_ = –0.321, *p* = 0.057), but no significant correlation between WM capacity and the proportion of APU stress assigned (*r*_S_ = 0.195, *p* = 0.254; see **Figure 2**). Note that these correlations are not completely complementary due to 5.9% unanalyzable trials.

## DISCUSSION

In sum, we observed a positive correlation of WM capacity with the proportion of APU stress assigned and a (complementary) negative correlation with the proportion of U stress assigned for non-words and a similar but non-significant pattern also for stress ambiguous words. There was no correlation of WM capacity with PU stress assignment. We would like to argue that this pattern of results speaks in favor of a leftward processing of word stress in German as participants with limited WM capacity only rarely produced the computationally most demanding (i.e., most leftward) APU pattern, while participants with good WM capacity were able to use APU stress. This interpretation is also supported by the increasing variance of APU stress assignment with increasing WM capacity: while participants with good WM were well able to assign APU stress, they were not restricted to that pattern.

More specifically, our observation that correlations were almost exclusively found for non-words with a closed final syllable is consistent with the assumption that tri-syllabic words with a closed final syllable are parsed into two metrical feet (a final non-branching foot and a preceding binary one) while words with an open final syllable are only parsed into one foot, leaving an unparsed initial syllable (Alber, 1997; Domahs et al., 2008; Knaus and Domahs, 2009). The two metrical feet, that words with closed final syllable are made of, offer two potential positions for main stress, while tri-syllabic words with open final syllable typically consist of only one metrical foot and an unparsed initial syllable, where main stress should be disfavored. In consequence, words with one metrical foot provide only one option of stress assignment, whereas words with two feet do require a decision were to place main stress, which may lead to increased processing costs. Indeed, non-words with open final syllable tended to attract less APU stress than words with closed final syllable, consistent with previous findings (Janssen, 2003b; Röttger et al., 2012; Domahs et al., 2014).

It may be argued that leftward stress computation leads to increased costs in speech production compared to rightward stress computation. Given that the sequence of phonemes is processed in a rightward manner, rightward stress processing is consistent with the processing direction of phonemes whereas leftward stress processing is inconsistent with it, causing elevated costs. Similar arguments have been put forward for left aligning vs. right aligning systems of secondary stress (Hayes, 1995; Alber, 2005). In left aligning systems, less phonological pre-planning may be required in speaking, given that the parser does not have to know the number of a word’s syllables before starting to assign stress. With respect to main stress, a look at the World Atlas of Language Structures Online (Goedemans and van der Hulst, 2014; features 14A and 15A) reveals that systems with right edge orientation are not rare. If, indeed, such systems are associated with increased processing costs, it remains an open question whether there is any compensation for this disadvantage in languages with right edge orientation including German.

If PU stress should be regarded as default option in German (Eisenberg, 1991; Wiese, 1996), one may have expected a processing advantage such that participants with limited WM capacity assign more (“default”) PU stress than participants with good WM capacity. Obviously, this was not the case. Previous studies have also failed to provide empirical evidence for a processing advantage of PU stress in German. PU stress was not the preferred pattern in violation paradigms (Domahs et al., 2008, 2013). Moreover, it was not dominant in monolingual (Röttger et al., 2012) or bilingual (Tappeiner et al., 2007) non-word production experiments either. Finally, PU stress was not the most robust pattern in cases of acquired language impairment (Janssen, 2003a; Janßen and Domahs, 2008). Clearly, we did not find evidence for a first syllable default either.

In sum, we would like to explain the present pattern of results based on cognitive procedures which operate in a leftward fashion to assign stress to non-words. These procedures may be sets of rules or constraints (Domahs et al., 2008; Knaus and Domahs, 2009), while we found no evidence for the psychological reality of a default stress position in German. For the first time, it has been demonstrated that the use of these procedures is influenced by individual cognitive processing capacity. At present, the procedures which are actually used during the computation of main stress remain unspecified. Nonetheless we suggest so far that (a) it seems to be more demanding to assign stress to a syllable (APU) which is distant from the starting point of these procedures (i.e., from the right edge of a word) than to a syllable (U) which is close to it and (b) this difference is more pronounced in non-words which contain two metrical feet compared to non-words containing only one foot. Future work should try to further elucidate the exact nature of stress assigning algorithms.

A processing-based account of stress assignment as sketched above could also explain the observation that two patients with impaired lexical knowledge due to primary progressive aphasia did not use any APU stress in cases of uncertainty (Janssen, 2003a; Janßen and Domahs, 2008). Given that both patients had a massively reduced WM span, APU stress assignment may have been too demanding for them. Their avoidance of APU stress has previously been explained with APU stress being exceptional and needing to be lexicalized (Knaus and Domahs, 2009). However, in the light of the present findings the processing-based account seems to be superior: It was the good participants who produced the largest proportion of the putative exceptional pattern and the participants with limited WM capacity who tended to avoid it.

We also found a near-significant negative correlation between WM capacity and the proportion of stress assignment to the final syllable for stress ambiguous words. This is remarkable given that stress assignment to German words is assumed to be fully lexicalized. In the case of stress ambiguous words, the two variants of a word (e.g., *Hórizont* vs. *Horizónt*) show a regional distribution, while individual speakers stick to one of the variants quite consistently, i.e., they have one lexical entry which is determined by their regional variant of German. However, the present results suggest that this is not the whole story. There was interindividual variance in stress assignment to those words which was systematically related to individual WM capacity while all participants were recruited in the same area (Aachen, Germany). In our view, there are two possible explanations for processing-related interindividual variance in stress assignment to ambiguous words: first, it may be that lexical retrieval of word stress in German has some form of right alignment. Thus, for some reason or another, the scanning of lexical entries for their stress information may occur in a leftward manner. Second, it may be that lexical retrieval co-occurs with the application of (rule-based) procedures as applied for stress assignment in non-words and that both processes interact. Given that at least one of the two processes (i.e., rule application) is more demanding for APU than for U stress, this may also influence lexical retrieval or the final articulatory output – at least in stress ambiguous words. Note, however, that all participants were well able to read existing unambiguous words correctly such that they produced merely any variance in stress assignment in such words.

Could the increasing use of ultimate stress with decreasing WM capacity be explained by factors other than the computational ease to place main stress on the final syllable? An alternative explanation may refer to articulatory preparation in general rather than to the computation of main stress position. According to this explanation, participants with limited WM span might tend to lengthen the final syllable of target (non)words to get more time for preparing the subsequent word and – given that duration is also a relevant phonetic cue to word stress – this might result in perceived stress on the final syllable. However, this potential artifact has been minimized in our experimental design as our target stimuli were embedded in carrier sentences/text, respectively. Recall that the carrier sentence was identical for all non-words, such that the word following the target was highly expectable and automatized during the experiment. Moreover, in non-word reading participants were instructed to first read the non-word silently and only if they felt ready to produce it fluently to utter the carrier sentence containing the target-non-word. Yet, to clear away the last doubts and to disentangle ultimate stress from final lengthening, further research may address languages with rightward stress assignment, where both effects would be separable.

Our data support accounts of German word stress, which assume leftward computation (e.g., Vennemann, 1991; Alber, 1997; Féry, 1998; Domahs et al., 2008), but seem to be at odds with those assuming rightward computation (Levelt et al., 1999). However, our data are silent with respect to the possibility that there are two co-phonologies of German with different stress computation directions (Wurzel, 1970, 1980; Benware, 1980; Féry, 1986). Given that we used tri-syllabic non-words in one task and that the stress ambiguous words used in the other task were all loan words, it seems plausible that both types of stimuli were treated within the “non-native” phonology by our participants. In this case, results of both tasks would lend support to the assumption that in this part of German phonology, stress computation proceeds in a leftward manner. Note that experimental evidence based on processing difficulty would be difficult to obtain for the other part of German phonology (native words), as this comprises mainly one- and two-syllable words which do not offer the possibility of “long distance” stress assignment.

In the remainder of this section we would like to argue that it is possible to reconcile the apparently contradicting assumptions on the direction of stress processing in German, based on a specification of the lemma model of speech production (Levelt et al., 1999). Recall that within this model, there are two stages of prosodic encoding: at the first stage (*frame generation*), a metrical frame is generated, specifying the number of syllables and the stress pattern of words (in case of non-default stress). At the second stage (*prosodification*), the sequence of segments is filled into the metrical frame and default stress is assigned. Note that the direction of stress processing is only specified for the prosodification stage, where rightward processing is assumed. There is no indication about the direction of stress processing at the frame generation stage. We would like to suggest that the assignment of stress to German words occurs during frame generation, given that there is no psycholinguistic evidence for a default stress pattern. At this stage, stress is computed in a leftward manner, i.e., APU stress assignment is more demanding than PU or U stress assignment. During prosodification, segments are filled into the metrical frame. According to Levelt et al. (1999), prosodification proceeds incrementally in a rightward manner. Therefore, the articulatory realization (but not the computation) of APU stress may be less demanding than the articulation of PU and U stress. This account is consistent with evidence from Italian, showing an articulatory advantage for stress positions at the left edge of a word: pseudowords stressed on the APU could be read faster than pseudowords stressed on the PU. On the other hand, the computation of main stress position for pseudowords was influenced from the phonological similarity with words on the right edge only (Burani and Arduino, 2004; Burani et al., 2013; Sulpizio et al., 2013). In other words: the processing direction between prosodification (starting at the left edge) and frame generation (starting at the right edge) may diverge. Furthermore, empirical evidence for rightward stress processing during the monitoring of lexical stress positions in Dutch reported by Schiller et al. (2006) seems to be related to the prosodification stage rather than to the frame generation stage.

Note that the lemma model is underspecified in several aspects of prosodic encoding. First, it does not incorporate the possibility of right aligning default systems (e.g., languages with fixed stress positions on the U, PU, or APU). Do the filling of the frame with segments (rightward) and the processing of default stress (leftward) occur in parallel or sequentially? Second, in some languages stress is assigned neither by default (fixed stress position) nor via lexical retrieval. Rather, it can be placed on variable positions, which are, however, determined by fully regular rules (e.g., Cairene Arabic or Latin). At which stage of prosodic encoding do these rules operate? A third point, which is related to the second one, concerns stress assignment to non-words. How can the assignment of stress to syllables other than the default be explained? Non-words are stressed on variable positions by German participants (Röttger et al., 2012). These positions are neither restricted to the default nor fully captured by rules. If the assignment of stress involves lexical analogies – are those retrieved during frame generation? Fourth, how can different processing directions for main and secondary stress (e.g., Alber, 2005) be incorporated into the model? Obviously, many further specifications of the model have to follow in the future. Yet, the main distinction into leftward stress processing at the frame generation stage and rightward realization of stress during prosodification could already capture a number of previously contradicting findings and theories on (German) word stress.

## Conflict of Interest Statement

The authors declare that the research was conducted in the absence of any commercial or financial relationships that could be construed as a potential conflict of interest.

## Footnotes

1 Here and in the remainder of this paper syllable structure only indicates vowels (V) and consonants (C) of the syllabic rhyme. Syllabic onset structure may vary and is therefore not specified.

2 Although perceptual judgment is the standard method in stress assignment experiments using production paradigms, this method does not allow disentangling the potentially distinct contribution of the different phonetic cues (e.g., pitch, duration, and intensity) which – in a complex interplay – lead to the perception of stress.
